# Quantitative analysis of zirconia and titanium implant artefacts in three-dimensional virtual models of multi-slice CT and cone beam CT: does scan protocol matter?

**DOI:** 10.1259/dmfr.20230275

**Published:** 2023-10-23

**Authors:** Ragai Edward Matta, Stephanie Knapp Giacaman, Marco Wiesmueller, Rainer Lutz, Michael Uder, Manfred Wichmann, Anna Seidel

**Affiliations:** 1 Department of Prosthodontics, University Hospital Erlangen of Friedrich-Alexander-Universität Erlangen-Nürnberg (FAU), Erlangen, Germany; 2 Institute of Radiology, University Hospital Erlangen of Friedrich-Alexander-Universität Erlangen-Nürnberg (FAU), Erlangen, Germany; 3 Department of Oral and Maxillofacial Surgery, University Hospital Erlangen of Friedrich-Alexander University Erlangen-Nürnberg (FAU), Erlangen, Germany

**Keywords:** artifact, zirconium, titanium, dental implant, three-dimensional imaging, computed tomography, cone-beam computed tomography

## Abstract

**Objectives:**

Artefacts from dental implants in three-dimensional (3D) imaging may lead to incorrect representation of anatomical dimensions and impede virtual planning in navigated implantology. The aim of this study was quantitative assessment of artefacts in 3D STL models from cone beam CT (CBCT) and multislice CT (MSCT) using different scanning protocols and titanium-zirconium (Ti-Zr) and zirconium (ZrO_2_) implant materials.

**Methods:**

Three ZrO_2_ and three Ti-Zr implants were respectively placed in the mandibles of two fresh human specimens. Before (baseline) and after implant placement, 3D digital imaging scans were performed (10 repetitions per timepoint: voxel size 0.2 mm³ and 0.3 mm³ for CBCT; 80 and 140 kV in MSCT). DICOM data were converted into 3D STL models and evaluated in computer-aided design software. After precise merging of the baseline and post-op models, the surface deviation was calculated, representing the extent of artefacts in the 3D models.

**Results:**

Compared with baseline, ZrO_2_ emitted 36.5–37.3% (±0.6–0.8) artefacts in the CBCT and 39.2–50.2% (±0.5–1.2) in the MSCT models. Ti-Zr implants produced 4.1–7.1% (±0.3–3.0) artefacts in CBCT and 5.4–15.7% (±0.5–1.3) in MSCT. Significantly more artefacts were found in the MSCT *vs* CBCT models for both implant materials (*p* < 0.05). Significantly fewer artefacts were visible in the 3D models from scans with higher kilovolts in MSCT and smaller voxel size in CBCT.

**Conclusions:**

Among the four applied protocols, the lowest artefact proportion of ZrO_2_ and Ti-Zr implants in STL models was observed with CBCT and the 0.3 mm³ voxel size.

## Introduction

Three-dimensional (3D) imaging enables precise spatial measurement, mapping of anatomical structures, and visualization of detailed bone structure.^1^ In addition to multislice CT (MSCT), cone beam CT (CBCT) is well established in dentistry because of its excellent delineation of small bony structures, relatively low costs, the option to apply low-dose protocols, and widespread availability.^2–6^ Regarding the 3D assessment of dental implant sites, CBCT is considered the imaging device of choice, as it offers enhanced visualisation of complex anatomical structures such as neurovascular channels and jaw bone.^7,8^ With the availability of computer-aided design (CAD) simulations and pre-operative treatment planning, the application of 3D imaging has led to a reduced intraoperative complication rate for dental implant placement.^9^

Despite many advantages, CBCT and MSCT imaging also have shortcomings, such as inaccuracies due to artefact formation.^10^ Artefacts are mostly caused by foreign materials—in the jaw area especially by crowns and bridges at the level of the masticatory plane. Once *in situ*, dental implants can also severely impair subsequent 3D radiological diagnostics by means of metal artefacts. These artefacts can arise for several reasons, and those induced by highly absorbing material in particular can lead to clinically relevant imaging errors. When emitted X-rays hit the metallic surface, they may be deflected from their original direction and reach the detector from an incorrect direction, leading to faulty data backprojection (*i.e*. “X-ray scatter”).^11,12^ This error is then reconstructed in the data set in proportion to the amount of scatter present in the tomography scan,^13^ displayed as highly intense streaks that also can affect the grey values of adjacent tissues.^12,14^ This influence can prevent correct assignment of the shape and density properties of metallic objects, like dental implants, as well as surrounding tissues and reduce diagnostic accuracy.^15^

For oral rehabilitation with dental implants, the combination of 3D radiologic imaging models, intra- and extraoral surface models, and CAD/computer-aided machine applications enable static computer-aided implant surgery (s-CAIS).^16^ Because 3D radiological imaging often does not display tooth surfaces with sufficient precision, the intraoral surfaces of teeth and attached gingiva can be integrated via intraoral surface scan or dental cast scan.^17^ First, the universal Digital Imaging and Communications in Medicine (DICOM) data from CBCT/MSCT are converted into 3D stereolithographic standard tessellation language (STL) models using segmentation. The STL models of CBCT/MSCT and intraoral scanner (IOS) then can be merged via their respective surfaces, *i.e*. the teeth, and analysis of the present anatomy of hard and soft tissues can be carried out.^18,19^ Various reasons for possible deviations from the virtually planned to actual implant position have been considered and investigated. One of the most important aspects, however, is the precision of the baseline data CBCT/MSCT scan for precisely registering the data and preventing transfer of errors to the surgical field.^19^

Implant planning software often offers an automatic conversion of DICOM to STL models with default grey value thresholds for the display of teeth and/or bony structures. However, artefacts can distort anatomical structures and reduce contrast between adjacent tissues.^20^ A software program’s automated algorithm cannot distinguish whether these grey values actually represent bone or artefacts, as both are rendered by high grey values/Hounsfield units (HUs) in CBCT and MSCT.^21^ The result can be unusable 3D data sets with surface values that deviate strongly from the actual situation. The falsely rendered surface of the MSCT/CBCT models consequently compromises the merging process with IOS scans. Currently, manual segmentation steps are still necessary and recommended to obtain optimal 3D models for digital case planning.^19^ It has been shown that with an increasing number of fixed zirconia crowns in the mouth, a significant deterioration of the matching accuracy can arise due to artefacts.^22^

In dental implantology, zirconium (ZrO_2_) is an alternative implant material in addition to the long-established material titanium (Ti) or titanium-zirconium (Ti-Zr). With respect to 3D digital imaging, zirconia implants show significantly more artefact formation in cross-sectional view in CBCT than titanium implants.^23^

For s-CAIS and in the context of virtual treatment planning, the CBCT or MSCT scan represents the baseline data. Given the importance and frequent use of 3D imaging in surgical planning, the aim of this study was to quantitatively investigate the influence of imaging modality and scanning protocol with different implant materials on image quality.

## Methods and materials

### Study design and specimen selection

**Figure 1.**
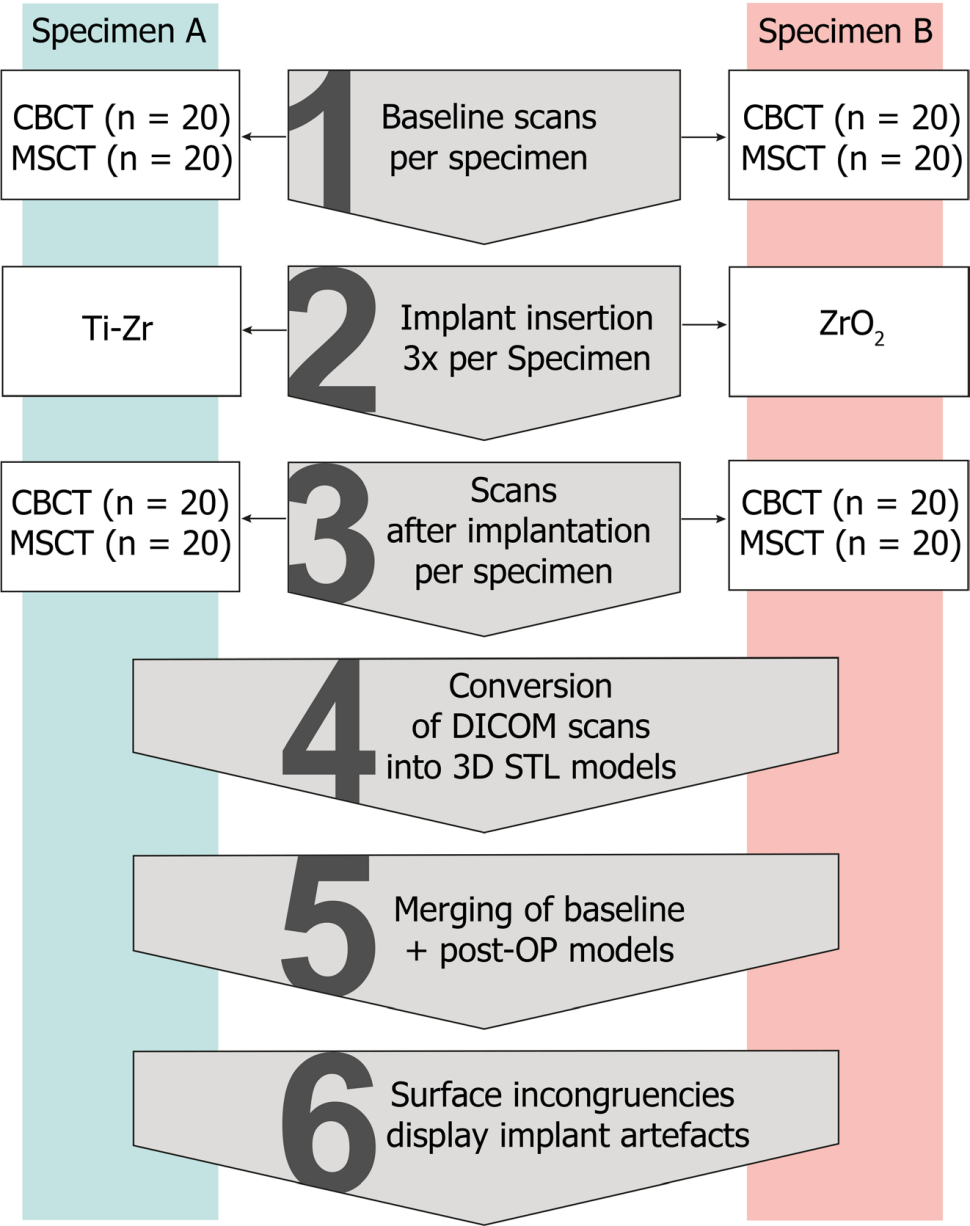
Step-by-step overview of the study design and workflow. 3D, three-dimensional; CBCT, cone beam CT; DICOM, Digital Imaging and Communications in Medicine; MSCT, multislice CT; STL, standard tessellation language.

With the design of this prospective comparative *in vitro* study, we aimed to recreate a situation that was as close as possible to the clinical *in vivo* situation. For this purpose, we used two human specimens, both provided by the Anatomical Institute of Friedrich-Alexander University Erlangen-Nürnberg after approval and according to the guidelines of the Medical Ethics Committee (Approval-Nr.: *126_15 Bc*). The criteria for the specimens were edentulousness, sufficient bone in the vertical and horizontal dimensions of the jaw, and no metallic restorations in the entire head region. Although a previous study showed that formalin fixation of the specimen has little influence on the accuracy of CBCT- and MSCT-derived 3D models, the cadavers used in this work were not embalmed and were in their natural fresh state.^24^ To achieve a high degree of standardisation, multiple repetitions of scans with the respective modality and setting were performed in the same two specimens (Figure 1). Regarding the two implant materials under investigation, the allocation of which specimen would receive the titanium Ti-Zr and which the ZrO_2_ implants was determined randomly. The focus was put on streak artefacts in automatically converted STL models generated from CBCT and MSCT data sets.

### Radiological data acquisition and implant insertion

To generate a radiological baseline data set that was free of metallic materials and their imaging influences, the specimens were first scanned in their natural state 20 times each in a CBCT scanner (3DeXam, KaVo Dental GmbH, Biberach, Germany) and 20 times each in a MSCT scanner (Somatom Definition AS, Siemens Healthcare, Forchheim, Germany) (Figure 1). Detailed acquisition settings are listed in Table 1. In the CBCT protocol, the kilovoltage setting was not variable, and scans were made at two resolutions 10 times each: 0.2 × 0.2 × 0.2 and 0.3 × 0.3 × 0.3 mm (voxel size 0.2 and 0.3 mm³). In MSCT, scans with two different kilovoltage settings were performed 10 times each: 80 and 140 kV.

**Table 1.** Overview of acquisition parameters used for MSCT and CBCT

CBCT					
Number of scans (baseline & post-op each)	Voltage [kV]	Effective mAs	Voxel size [mm]	Field of view [mm]	Acquisition time [s]
*n* = 10	120	18.54	0.2 × 0.2 × 0.2	160 × 120	8.9
*n* = 10	120	18.54	0.3 × 0.3 × 0.3	160 × 120	8.9

MSCT									
Number of scans (baseline & post-op each)	Voltage [kV]	Effective mAs	Slice thickness [mm]	Field of view [mm]	Convolution kernel	Pitch factor	Feed per rotation [mm]	Acquisition time [s]	Mean CTDIvol [mGy]
*n* = 10	80	100	0.75	212 × 212	H70	0.9	34.5	4.68	4.36
*n* = 10	140	100	0.75	212 × 212	H70	0.9	34.5	8.16	21.41

CBCT, cone beam CT; MMSCT, multislice CT.

After the baseline scans were completed, implant insertion was conducted by a senior dental implantologist. One of the specimens was randomly selected to receive three Ti-Zr implants (85% titanium and 15% zirconium) (Roxolid SLActive Standard, Straumann, Basel, Switzerland) with a diameter of 4.1 mm and length of 12 mm. Three zirconia ceramic implants (100% yttria-stabilized zirconia; PURE Ceramic, Straumann) with a diameter of 4.1 mm, length of 12 mm, and abutment height of 4.0 mm were placed in the second specimen. With implant bed preparation completed according to the manufacturer’s recommendation, three implants per specimen were placed interforaminally in the mandible with sufficient stability (Figure 1).

Immediately after implant placement, a second scanning cycle (post-operative/post-op) was performed for CBCT and MSCT, respectively, using the identical technical parameters and repetitions as for baseline data (Table 1, Figure 1).

### Data conversion and 3D software-based evaluation

The data derived from CBCT and MSCT scans were converted from the DICOM format to STL by applying default window settings for bone surface display (range: 150–2000 HU) in medical imaging software (Impact View 4.4.1, CT Imaging, Erlangen, Germany). Identical window settings were applied for all image data. The software-based evaluation was performed by one experienced and well-trained operator.

Subsequently, STL bone models were imported into a CAD analysis software (Gom Inspect 2020, Gom GmbH, Braunschweig, Germany). Each data set was trimmed in the same way with the pogonion as the bone reference, resulting in a 3D model of the mandible. For analysis of the surface deviation between baseline and post-op, models representing the specimens’ baseline state were set as the reference value and then each superimposed with one model of the post-op state containing implants (actual value) (Figure 1). The respective baseline and post-op models were merged in a two-step process: first a manual superimposition via prominent bone structures as reference was performed (GOM Inspect, Gom GmbH). Then, the software’s “local best-fit” algorithm was applied in order to achieve highly precise, software-based merging results for the digital investigation.^25,26^

**Figure 2.**
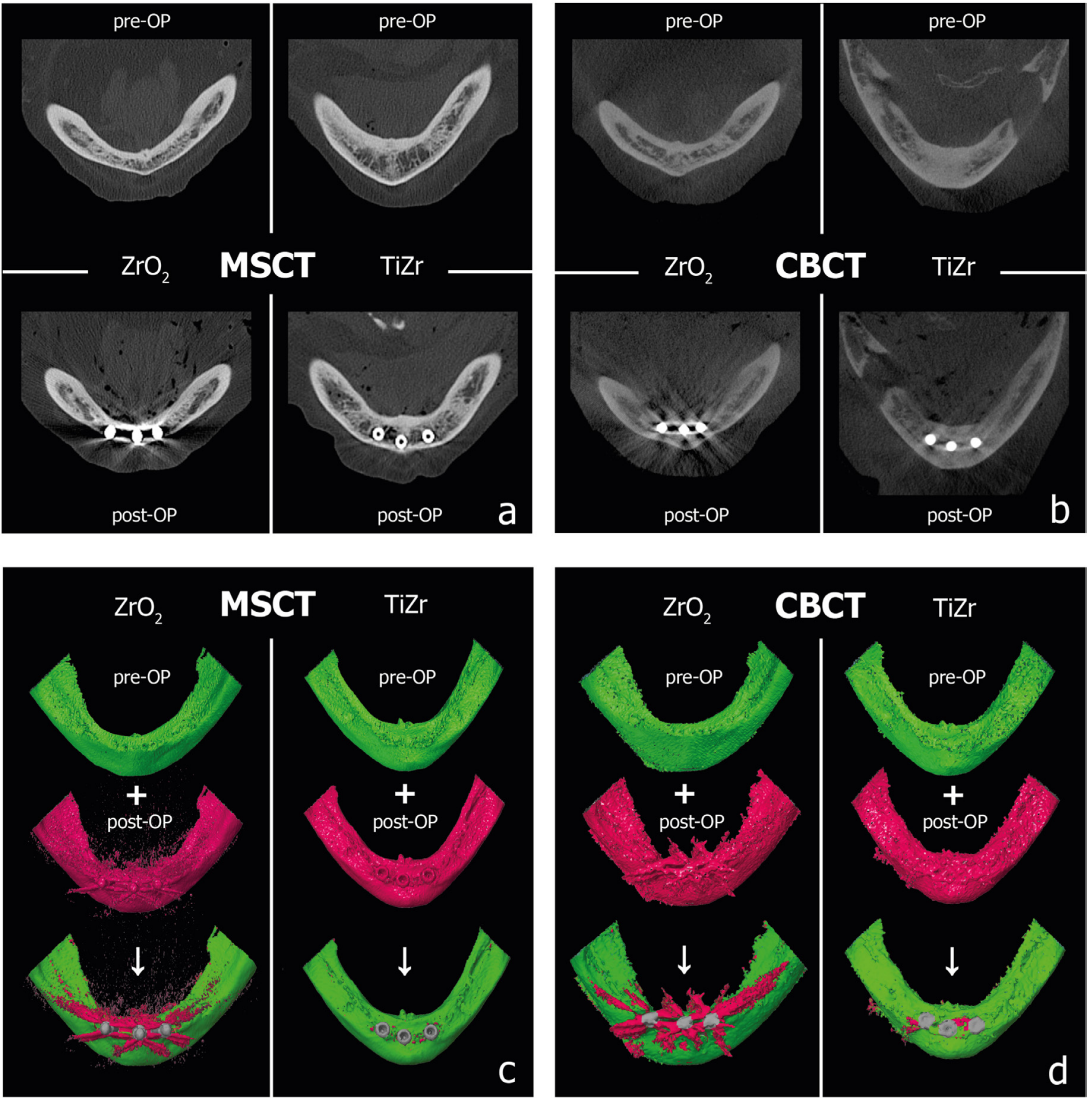
**(a**) Two-dimensional sectional view of the baseline and post-op scans with ZrO_2_ and Ti-Zr implants in the mandible in MSCT and (b) in CBCT. (**c**) Merging process of the corresponding STL models with 3D representation of the baseline models (green) and the artefact containing post-op models (red) for MSCT and (d) for CBCT. CBCT, cone beam CT; MSCT, multislice CT; STL, standard tessellation language.

Subsequently, a surface comparison between the actual and reference models was performed.^25^ This comparison enabled the automated, quantitative calculation of artefacts as a percentage of the total surface area of the mandibular model compared with baseline (Figure 2). After the merging process, the region of interest was determined, which was exactly the same size for all models, with the pogonion as the centre. The implants were manually deselected applying auxillary geometries—uniform cylinders with the exact diameter and length were designed so that the surface comparison included only the potentially present artefacts and not the implant bodies themselves (Figure 2). Incongruent areas of the actual and reference models that exceeded a tolerance limit of 500 µm were calculated as a percentage.

### Statistical evaluation

The percentage surface deviations from post-op to baseline data sets for MSCT and CBCT models with titanium and zirconia implants were summarised as descriptive statistics with mean value, standard deviation, and minimum and maximum values (Table 2). Statistical evaluation was performed using R, v. V 3.4.3 (R Foundation for Statistical Computing, Vienna, Austria).^27^ For pairwise comparisons of the device used (MSCT *v*s CBCT), the applied scanning parameters (80 kV *v*s 140 kV for MSCT and voxel size 0.2 *vs* 0.3 mm^3^ for CBCT), and implant materials (ZrO_2_ *vs*. Ti-Zr), Mann–Whitney *U* tests were performed (Table 3). A value of *p* < 0.05 was considered to indicate statistical significance. The Benjamini–Hochberg correction for multiple testing was applied.

**Table 2.** Descriptive analysis of calculated surface discrepancies from baseline

Implant material	Device	Group	Number of scans (baseline & post-op)	Mean [%]	SD [%]	Min [%]	Max [%]
ZrO_2_	MSCT	80 kV	***n* = 20**	**50.18**	±1.21	47.9	51.7
		140 kV	***n* = 20**	**39.17**	±0.53	38.6	40.1
	CBCT	0.2 mm³ voxel	***n* = 20**	**37.28**	±0.76	35.9	38.2
		0.3 mm³ voxel	***n* = 20**	**36.53**	±0.58	35.9	37.9
Ti-Zr	MSCT	80 kV	***n* = 20**	**15.71**	±1.28	14.0	17.8
		140 kV	***n* = 20**	**5.39**	±0.45	4.9	5.9
	CBCT	0.2 mm³ voxel	***n* = 20**	**7.13**	±3.03	4.2	10.9
		0.3 mm³ voxel	***n* = 20**	**4.08**	±0.35	3.6	4.7

CBCT, cone beam CT; MSCT, multislice CT; SD, standard deviation.

**Table 3.** *p-*values of the compared groups for implant artefact amount

Group	Test	Mode	*P*
Material	ZrO_2_ *vs* . Ti-Zr	MSCT 80 kV	< 0.001
	ZrO_2_ *vs* . Ti-Zr	MSCT 140 kV	< 0.001
	ZrO_2_ *vs* . Ti-Zr	CBCT 0.2 mm³ voxel	< 0.001
	ZrO_2_ *vs* . Ti-Zr	CBCT 0.3 mm³ voxel	< 0.001
Device	MSCT *v*s CBCT	ZrO_2_	< 0.001
	MSCT *v*s CBCT	Ti-Zr	< 0.001
Settings MSCT	80 *vs* 140 kV	ZrO_2_	< 0.001
	80 *vs* 140 kV	Ti-Zr	< 0.001
Settings CBCT	0.2 *vs* 0.3 mm³ voxel	ZrO_2_	0.045
	0.2 *vs* 0.3 mm³ voxel	Ti-Zr	0.001

CBCT, cone beam CT; MSCT, multislice CT.

## Results

The scattering artefacts emanating from the implant materials Ti-Zr and ZrO_2_ in two different human specimens were quantitatively determined by calculating the percentage of surface deviation of the post-op model (with implants) from the virtual baseline model (natural state) (Figure 2). Table 2 gives detailed results for MSCT and CBCT, scan protocol, and implant material.

### 3D diagnostic device

**Figure 3.**
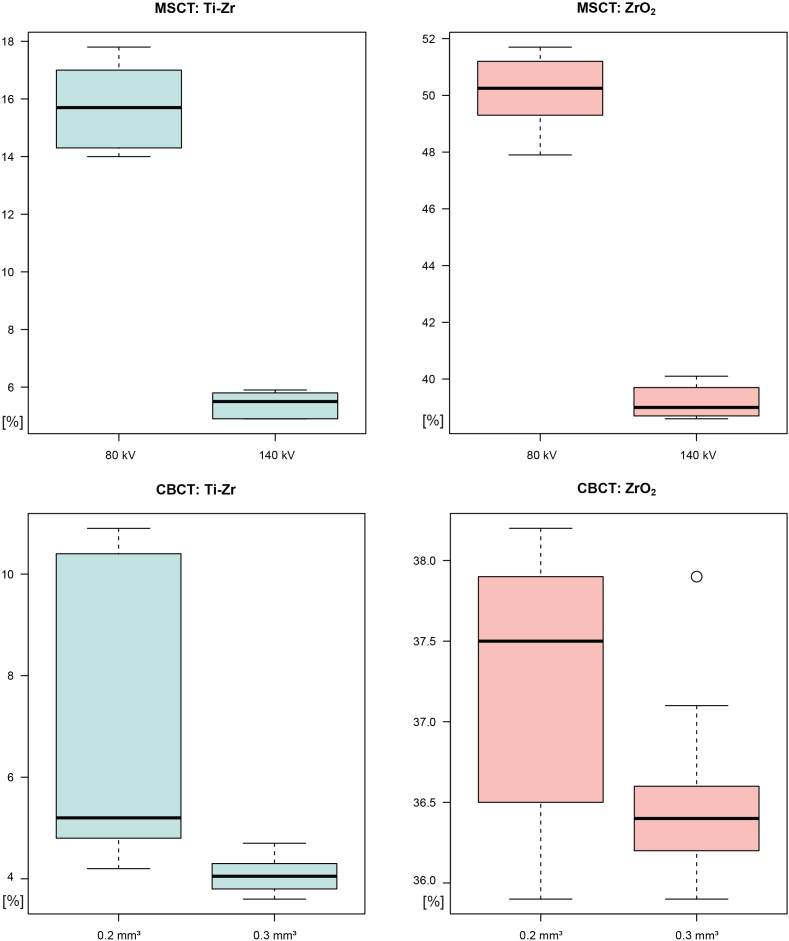
Boxplots displaying the summarized artefact quantity in MSCT and CBCT tested on ZrO_2_ and Ti-Zr implants with different acquisition settings. CBCT, cone beam CT; MSCT, multislice CT.

For the two 3D diagnostic devices used, the CBCT models presented significantly lower proportions of artefacts than MSCT for both materials under investigation (Table 3). For CBCT, the mean values of artefacts encountered for Ti-Zr were 7.1–4% compared with baseline and 15.7–5% for MSCT (Figure 3, Table 2). The comparison of CBCT (voxel sizes 0.2 and 0.3 mm^3^) and MSCT (80 and 140 kV) showed significant differences for both implant materials (Table 3).

### Acquisition settings

For MSCT settings, with higher tube voltage, significantly fewer artefacts were produced for both materials tested (Figure 3, Tables 2 and 3). In the specimen with Ti-Zr implants, at 80 kV, artefacts were three times more common than at 140 kV (15.71% to 5.39%, respectively). For the ZrO_2_ implant MSCT models, applying 140 kV led to 20% less artefact compared with the 80-kV scans (39.17% to 50.18%, respectively).

Regarding the CBCT settings applied, the change in voxel size from 0.3 to 0.2 mm³ resulted in slightly more artefacts in the ZrO_2_ implant models (from 36.53 to 37.28%) (Table 2). Percentages for the Ti-Zr models were 7.13 and 4.08% for 0.2 and 0.3 mm³, respectively.

### Implant material

Regarding Ti-Zr *v*s ZrO_2_, Ti-Zr was associated with significantly fewer artefacts in 3D models for all tested groups (Table 3). The highest quantity of artefacts occurred for ZrO_2_ implants in MSCT scans with 80-kV tube voltage (50.18±1.21%).

## Discussion

For three decades, the steps leading to oral rehabilitation with implants have become increasingly digitised, and this trend is expected to continue. To avoid diagnostic errors using 3D devices, the operator should be familiar with the acquisition technique and be able to deal with challenges that may arise from imaging artefacts. Achieving these aims can be difficult when metal artefacts are present, *i.e.* the buccal bone adjacent to implants may be underestimated by 0.22–0.27 mm because of material blooming effects in sectional views.^28^ Therefore, assessing peri-implant bone tissue in CBCT as a standard diagnostic tool is not recommended, and use of conventional 2D radiography is suggested instead.^29^ In particular, converting CBCT data into 3D models involves some pitfalls, most notably from metal artefacts when zirconia or titanium implants are *in situ*, and distortion, information deletion, and surface alteration of the 3D data may occur.^12^ When this falsely depicted surface model is further used for s-CAIS, backward treatment planning, or similar, the result can be significantly reduced alignment accuracy with intraoral 3D models^30^ and a direct negative influence on treatment.^19^ Because of these potential pitfalls, we investigated the effects of choice of implant material and acquisition settings of routinely used imaging modalities (CBCT and MSCT) on quantity of metal artefacts in 3D STL models.

In our study, ZrO_2_ and Ti-Zr implants led to differences in artefact quantities in the generated STL models. We also found a significant difference between CBCT and MSCT in artefacts in 3D models, and with regard to the setting parameters in MSCT (Table 3, Figure 3). The surface deviation of the generated 3D models with implants compared with baseline was close to 50% greater (ZrO_2_) and 20% greater (Ti-Zr) in the MSCT than CBCT models. Models derived from CBCT exhibited significantly fewer artefacts for both implant materials investigated (Tables 2 and 3, Figure 3).

To date, there is limited evidence of artefact quantity in 3D-generated stereolithic models of CBCT and MSCT using human specimens with different implant materials. Most studies have evaluated metallic artefact quality in cross-sectional views in 3D imaging. For instance, one method involves image quality evaluation in the presence of metallic artefacts using the contrast-to-noise ratio, as applied by Kursun-Cakmak et al.^31^ Their results showed that scans containing titanium implants had higher contrast-to-noise ratios and thus better image quality than scans with zirconia implants. Furthermore, metallic artefacts were more pronounced in MSCT than CBCT.^31^ These results could be confirmed in our analysis for 3D models. Another method of analysis of metal artefacts is the comparison of attenuation values via mean grey values.^23,32–34^ Demirturk Kocasarac et al, *e.g.* investigated the artefacts of the different implant materials in CBCT located within the field of view and outside, *i.e.* in the exomass. They found the greatest artefact expression in CBCT in terms of heterogeneous grey values with zirconia implants compared with Ti-Zr and titanium.^33^ Sancho-Puchades et al found that zirconium implants generated significantly more artefacts.^23^ One exception to this pattern is the study by Smeets et al, who found that in MSCT, zirconia implants had significantly greater effects on HU values and artefact intensity, whereas in CBCT, Ti-Zr artefacts were most severe, followed by zirconium and finally titanium.^34^ In our study, the artefacts were investigated for implants located within the field of view. The effect of significantly higher artefact levels for zirconia implants in 3D STL models was confirmed. Vitulli et al found in their comparison of the two materials (titanium and zirconia) with regard to the accessibility of the adjacent trabecular bone, that only ceramic implants had an adverse effect because of their strong artefacts.^35^

The reason for the high artefact emission of zirconia is the material’s high atomic number and corresponding absorption coefficient.^12^ In addition, various other factors can influence the general quality and artefacts in 3D digital imaging data. Regarding the accuracy of 3D STL models from MSCT compared with CBCT, conventional MSCT has been shown to provide higher accuracy.^24^ In the current study design, MSCT was conducted with higher tube current (mAs effective) (Table 1), which generally results in a reduced noise level.^12^ However, a significantly higher percentage of artefacts was produced in MSCT compared with CBCT for both implant materials investigated (Tables 2 and 3). In the present study, the automatically segmented 3D model surface was considered but not grey values in sectional images. Furthermore, previous studies have shown that operating scans with a higher kilovolt setting in MSCT can lead to a reduction in metal artefacts.^11,36^ The described starvation of the X-ray tube-emitted photons is caused by high-density objects like titanium or ceramic implants and leads to artefacts such as scattering and beam hardening after image reconstruction. With higher tube voltage, the average photon energy increases and attenuation is reduced. This effect is seen in the current results: the models with the 80-kV MSCT data showed the most artefacts for each material, with 50.2 and 15.7% for ceramic and titanium, respectively.

In this study, significantly more artefacts were observed in MSCT than in CBCT, regardless of implant material. These results are consistent with the observations of Hirschinger et al,^37^ with CBCT set at a non-variable tube voltage of 120 kV, producing about 13% less artefact for ceramic and up to 50% less for titanium implants compared with MSCT. However, when looking at the current results with increased voltage of 140 kV for MSCT, they are within the range of CBCT artefacts (Table 2). Although CBCT seems to be superior to MSCT, it must be noted that both modalities involve different acquisition techniques.^38,39^ When comparing the settings of 0.2 *v*s 0.3 mm³ voxels in CBCT regarding the present artefacts, significant differences were seen with both materials, although not as pronounced as for the other variables (Table 3). In the CBCT models, the 0.2 mm³–voxel setting produced slightly more artefacts (37.3% for ZrO_2_ and 7.1% for Ti-Zr) than the 0.3 mm³–voxel setting (36.6 and 4.1%). In another study by Dach et al, the accuracy of 3D models generated in the CBCT was higher for 0.2 mm³ voxel size, but nevertheless the 0.3 mm³-voxel models fell within the clinically acceptable range with a deviation of 0.33–0.22 mm.^40^ Accordingly, for implant planning and 3D models with implants *in situ*, a voxel size setting of 0.3 mm³ is sufficient and even to be preferred with regard to artefact occurrence.

Overall, the methods for measuring artefacts in 3D digital imaging are inhomogeneous, as are the study designs. Some studies have used human specimens, others plaster or gelatine models.^23,34^ To simulate the clinical situation as accurately as possible, we chose human specimens.^24^ It should be noted that this study was carried out on two fresh specimens with soft tissue. To achieve greater power and significance from a clinical point of view requires a more extensive *in vitro* study with a larger number of samples. Nevertheless, with the current study design, an objective statement can be made about the quantity of artefacts produced in 3D STL models.

Regarding the clinical perspective, the results suggest which device and which settings allow for reductions in the occurrence of metal artefacts in 3D models for the two different implant materials. If the CBCT/MSCT data set has not been corrected by an artefact-reduction algorithm before backprojection, options are limited for avoiding conversion of the interfering artefacts into the 3D data set, apart from time-consuming and error-prone hand segmentation.^12^ Metal artefact reduction in radiological data needs to be addressed in further research because 3D diagnostics and digital treatment planning are expected to increase with the constant development of better CBCT devices with less radiation exposure and more patients being restored with implants. Recent studies have investigated the possibilities for minimizing the occurrence of image errors and demonstrated that with the integration of deep learning and artificial intelligence, image quality of 3D data sets can be improved.^32,41–43^ This technique is predicted to be feasible and applicable to 3D imaging devices in the near future.^43^ Although great progress has been made in this field, current implant and surgical planning software cannot yet sufficiently and automatically remove implant-emitted artefacts in the process of recalculating and converting data into 3D models.^19^

The importance of the CBCT/MSCT scan as a basic data set for s-CAIS is evident. If data errors occur in this first step of planning (the scan), there can be subsequent effects on the clinical outcome: in digital workflows, the applied merging process is more precise with the STL models from CBCT/MSCT without artefacts.^19^ Without interfering artefacts, the surface representation in the 3D STL model can be in a clinically acceptable range of <300 µm if suitable setting parameters are selected in the CBCT.^17,40^ The current results highlight which device and parameter settings provide optimal 3D models for zirconium and Ti-Zr implants in terms of artefact quantity.

For this study, only one CBCT device was available, so the findings may not be extrapolated to CBCT scanners from other manufacturers given the potential variation in the resulting scans. Because of the heterogeneity of scanning parameters and devices, it is not possible to provide clear, universally applicable recommendations that will not quickly become obsolete among the ~279 CBCT devices on the market and a rapidly evolving technology.^44^ With the advanced techniques available and rapidly developing in MSCT and CBCT, it would be interesting in terms of further research to determine whether artefact quantity and quality of the 3D STL models are influenced by, *e.g.* artefact-reduction algorithms or low-dose protocols.

## Conclusion

With this study, the respective quantity of Ti-Zr and ZrO_2_ implant artefacts in 3D STL models could be identified and compared by radiological device (CBCT and MSCT) and acquisition settings. Within the limitations of the study, the following conclusions can be drawn:

1. Both implant materials produced artefacts in the 3D STL model, with zirconia emitting quantitatively more than Ti-Zr.
2. The artefact quantity in 3D models can be reduced by higher tube voltage settings in MSCT.
3. For CBCT, a voxel size setting of 0.3 mm^3^ is associated with the lowest artefact values for both implant materials and is clinically acceptable for generating models for implant planning.
